# Mapping the landscapes of the Stalinist mass repressions

**DOI:** 10.12688/openreseurope.14410.1

**Published:** 2022-04-07

**Authors:** Judith Pallot, Sofya Gavrilova

**Affiliations:** 1 Aleksanteri Institute, Helsinki University, Helsinki, Usimaa, 00170, Finland; 2 Leibniz Institute for Regional Geography, Universität Leipzig, Leipzig, 04109 Leipzig, Germany

**Keywords:** Stalin, Repressions, Historical Geographic Information Systems (HGIS); Landscape, USSR, Gulag

## Abstract

In this article, we focus on the ways in which a variety of different carceral techniques used to punish and exploit people’s labour during the Stalin period (1927—1953) in the Union of Soviet Socialist Republics (USSR) created a distinctive landscape of repression. Using the tools of historical geographic information science (GIS) to map the material landscape, we foreground space in the discussion of the USSR’s exceptional history of repression. The ‘carceral conditions’ frame allows us to deconstruct boundaries erected over more than half a century of writing the history of the USSR that have maintained artificial distinctions between the victims and impacts of different punishment modalities.  In the article, we follow the example of the Stanford Holocaust Geographies Project in combining quantitative and textual data with the spatial analytical tools of geovisualisation to reveal the patterns of events as the Stalinist repressive apparatus extended its reach across Soviet space.  In fixing the geolocation of carceral institutions and layering the resultant pattern with different types of qualitative and quantitative information in the same visual space, we hope to counter some of the myths and generalizations that exist in the literature about the geography of Soviet gulag. We use the case study of Perm’ region in the Urals to highlight the spatiality of the production of the material landscape of repression in one region.  Our aim is to position the USSR in the now substantial geographical literature discussing the twentieth century history of crimes against humanity and genocide and to suggest to historians that the geovisualisation of data can add a new dimension their studies of the Stalin period.

## Plain language summary

There are a many popular assumptions about how people were punished in the Soviet Union and today in the Russian Federation, including, for example, the idea that offenders have always been ‘sent to Siberia’. In fact, the patterns of punishment and repression in the Soviet Union were complex and highly differentiated. In this article we use the technique of geographical information system (GIS) mapping to demonstrate the ways in which the history of the Soviet repressions under Stalin unfolded in space and time. Typically, maps of the gulag and deportations in mid- century USSR are highly generalised and can mislead. Historians are new to the use of GIS but, as we show in this article, it can be applied in different ways both to quantitative and qualitative archival and other historical data. Applying GIS to existing and new data about the Stalin repressions allows the visualization of processes that cannot be captured easily in a print format to open up new lines of enquiry. We are mainly interested in this article is showing how the landscape was transformed by the various elements of the Soviet penal and disciplinary system into to what we identify as the ‘landscape of repression’. We do this for three spatial scales: firstly, for the national scale showing how the gulag unfolded in space and time across the USSR and secondly, at the regional scale showing how different ‘punishment modalities’ created a distinctive settlement pattern. Thirdly, using the biography of a single victim of peasant deportation from the western borderlands, we show how individuals traversed the landscape of repression at a local level. Mapping, at any of these scales, can help dispel some of the popular myths about the landscapes of repression that still have currency in public discourse, inside and outside Russia.

## Introduction

The archipelago metaphor often surfaces in academic discourse about the theory and practices of punitive confinement. The metaphor’s attraction for geographers is its association with the early works of Michel Foucault, who used it in his writing on power/knowledge to describe the penetration of the social body by technologies perfected in the prison, as a group of physically dispersed yet inter-related islands that “covers the entirety of society” ( Foucault *et al.,* 1980). Alexander Solzhenitsyn writing in the first edition of *the Gulag Archipelago, 1918—1956: An Experiment in Literary Investigation*, had introduced the archipelago metaphor when describing Stalinist labour camps in the north-east of Siberia;

“And the Kolyma was the greatest and most famous island, the pole of ferocity of that amazing country of Gulag which, though scattered in an Archipelago geographically, was, in the psychological sense, fused into a continent—an almost invisible, almost imperceptible country inhabited by the zek people [convicts]. And this Archipelago crisscrossed and patterned that other country within which it was located, like a gigantic patchwork, cutting into its cities, hovering over its streets” ^1^.

An invisible island chain separated from the mainland is a fitting metaphor for the isolation of penal confinement associated in the public imagination with the celebrated island prisons of Van Diemen’s land, French Guiana, the Tuscan archipelago, or San Francisco Bay. The metaphor has also been put to the service of the one-way journey to the concentration camps of the Third Reich, and the re-education camps of Xinjiang Uygur and North Korea. For the inhabitants of Kolyma, and other northern camps, the rest of the Russian landmass was ‘the mainland’. 

In academe, the archipelago metaphor was given endorsement in the 1960s by sociologist Erving Goffman’s concept of the total institution, which he defined as “a place of residence and work where a large number of like situated individuals, cut off from the wider society for an appreciable period of time, together lead an enclosed, formally administered round of life.”( Goffman, 1961: xiii). Goffman’s classic text concerned psychiatric asylums but his insights were rapidly taken up by others studying closed institutions. Research in penal sociology since Goffman has revealed the 21st century prison to be far more permeable and less enclavic than the archetype, and this is true both for penal institutions to which inmates have been sentenced by extra-legal processes and to penal institutions in rule-of-law governed states. The labels gulag, neo-gulag, penal, and carceral archipelago are now applied more broadly to the less-than-total total institutions of the Russian penal system under Putin, the US supermax and Guantanamo, and Western prisons. ^2^

As for the ‘gulag proper’, revisionist histories drawing on declassified archives have also shown that the Soviet gulag’s facilities were permeable; non-convoy prisoners worked alongside what was described as voluntary labour on major construction projects from the 1930s and the revolving door circulated individuals between freedom and captivity into the late Soviet period. In the wake of these findings, Solzhenitsyn has been criticised for his poor choice of geographical imagery, but in his defence, metaphors are poetic colourations or elaborations of an idea and are not supposed to be taken literally. More troublingly, Solzhenitsyn is often now dismissed as an unreliable witness. ^3^ In the Soviet version of Holocaust denial, Russian historians have to demonstrate their patriotism by holding Solzhenitsyn to account for exaggerating the negative features of the gulag. There are very few historians in the Russian Federation working on the gulag at the present time for fear of falling foul of recent legislation criminalising the falsification of the motherland’s history ( *Novaya Gazeta*, 10/09/20ot the idea of the gulag from the French system of relegation ( Plamper, 2002: 266). But these criticisms overlook Foucault’s thesis that the techniques perfected in the prison spread out into the whole social body. Viewed through the Foucauldian lens, with its emphasis on discourse, knowledge, power, government and subjectivity, more nuanced questions surface about the spatiality of Soviet penality than typically are found in historical accounts of the gulag. It is not our intention here to discuss the full range of concepts that Foucault or his followers offer gulag scholars but rather it is to bring his insights on the transformative role of power flowing through space to the centre of analysis of the history of Russian penality. This fits the agenda of carceral geography, with its focus on the lived-experience, geographical distribution, and interconnectedness of spaces of confinement ( Moran, 2015). In carceral geography, the object of study is not the prison *qua* penal institution, but the ‘power-filled and socially expansive’ understanding of the carceral that incorporates the different situations that cause people harm as a result of their confinement and restrictions on their mobility ( Moran *et al.,* 2018).

Carceral geography’s understanding of the carceral is helpful in describing the situation in Stalinist’s USSR. At one end of the carceral spectrum were sites of interrogation and summary execution in the cellars of metropolitan prisons, and at the other there were so-called special operations — classic gardening episodes that expelled ‘hooligan’ elements to 101st kilometre beyond selected cities. ^4^ Between these poles, were myriad categories and sub-categories to which people were assigned by virtue of their actions and inactions, ethnicity or race, class position, and social relationships, and that, in turn, determined to which disciplinary and punitive measures they were subject. From 1929 to 1953, the state elaborated ever finer functional and social classifications of the ‘special contingent’ or *spetskontigent*. This was the label attached to anyone on the receiving end of the state’s punitive measures and it remains in use today by officials, scholars, and other commentators when referring to convicted offenders. 

The menu of punitive measures available to power and the complex taxonomy of individuals and social groups to which these were applied, makes writing the history of the carceral conditions of the Soviet repression challenging. Historians have tended to concentrate their research firmly within the cells of the Soviet-era taxonomic matrix of whom should be punished or disciplined, and how. The taxonomy today has acquired near paradigmatic status among an older generation of scholars, resulting in the neglect of some lines of enquiry. For example, it is rare to find the question of race, as opposed to class, raised in discussions of the everyday experiences of prisoners in the gulag. The reasons for this are practical, relating to how data were collected, stored, and are accessed today, but they also reflect scholarship’s internalisation of the *a priori* assumptions that informed Soviet-era ideologies. This has impacted the broader debates about where we position the Soviet repressions in relation to the twentieth century history of crimes against humanity and genocide. 

No less paradigmatic in scholarship on the Stalin repression is the strong distinction that, typically, is drawn between static and mobile carceral modalities. The longevity of penal transportation in Imperial Russia and its continuation after the 1917 Revolution has contributed to exile being viewed as a discrete disciplinary form, separate from the punishment of imprisonment. In fact, the mobile/static binary is an artificial construct, as both modalities are constitutive of the other. This point is argued in Piacentini & Pallot (2014) in our conceptualisation of ‘in exile imprisonment’ to describe the centuries-long predilection in Russia for the peripheries to be used as places of punishment. The importance of displacement and the “gathering in” of *homo sacer* to the camp, has been underlined by Derek Gregory (2019) in developing his critique of Agamben ( Gregory, 2006: 20–211). In the 21 ^st^ century, prisoners in Russia continue to be subjected to long journeys during which they experience punishment’s familiar pains ( Moran *et al.,* 2012). Notwithstanding these challenges to the mobile/static punishment binary, gulag scholarship tends to follow the traditional dividing lines between the punishment of being sentenced to forced labour in a penal facility or the punishment of being sent into exile.

A consequence of adherence to the mid-twentieth century Soviet taxonomies, whether of punishment forms or the targeted individuals and groups, is, thus, a fragmented history of the mass repressions that tends to overlook the intersectionality of class, race, ethnicity, and gender among its victims. This fragmentation of scholarship is compounded by the tendency for researchers to stay firmly within the established periodization of Soviet and Russian history that equates dominant punishment modalities with successive Soviet leaders. Consequently, it is as rare to find research that considers punishment forms diachronically. There is clearly abundant scope for more critical engagement with Russia’s violent history. In what follows, we use the term repression to describe the various carceral forms that bore down on people as a result of the legal, social, and political sanctions they were subjected to in the Stalin era. This term is preferable to ‘gulag’ which is popularly used as shorthand for all Stalin’s repressive measures against the Soviet people but is a source of confusions and exclusions. Here, we follow the example of historical scholarship reserving the use of gulag exclusively for the forced labour camps, and certain other specified institutions that fell under the jurisdiction of the Chief Administration for Camps ( *Glavnoe Upravlenie Lagerei*) that was located within or constituted the internal affairs ministry (OGPU-NKVD-MVD) ^5^ from 1930 to 1960. We are not just avoiding terminological confusion, however. In focusing on the mass repressions, we are freeing ourselves to take the broadest possible approach to the transformations punitive measures taken against the population in the Stalin era effected in the built and natural environment, and on the lives of the agents and victims of these changes. 

It is a sad comment on geographical scholarship that Russia remains beyond its vision. This, no doubt, is because of the hostile environment for research and linguistic barriers, but it is also because of the post-colonial construction of Russia as a “subaltern empire in a Eurocentric world” ( Stuenkel, 2015). ‘Western’ geographers have been all too ready to marginalise Russia compared with other world regions ( Müller & Trubina, 2020; Trubina *et al.,* 2020). Given the scope of Putin-Russia’s geopolitical interventions, we find this difficult to understand. The scholarly deficit is particularly obvious in the large body of geographical scholarship that exists on violent, terror and lethal landscapes. In these studies, the European lineage is traced back to the Holocaust, about which there is now a substantial literature on the philosophical and historical-geographical issues, as well as detailed empirical studies. By contrast, the Soviet mass repressions, which were longer-lasting and had a greater total death toll are, at best, a marginal add on. When the gulag does appear alongside Nazi concentration camps in discussions of ‘spaces of exception’, it is typically without reference to the critical scholarship that has arisen recently challenging the ontological assumptions, described above. Revisionist scholarship has examined varied spaces produced by the mass repressions and our historian colleagues have engaged with the theorizations of Agamben, Foucault, and Le Febvre to explain them ( Barenberg & Johnson, 2022; Barenberg, 2014; Barnes, 2011; Baron, 2012; Bell, 2013; David-Fox, 2016; Hardy, 2012). New fruitful avenues of research have also been identified in dialogue with penal sociologists. Penal sociology provides scholars with the tools needed for both diachronic and geographical comparison of the ‘conditions of carcerality’ and have already opened up new perspectives in the analysis of penality in the Soviet and post-Soviet sphere (see, for example, Moran *et al.,* 2012; Pallot, 2015a and Pallot, 2015b; Pallot & Piacentini, 2012).

In this article, we aim to bring the Soviet mass repressions in from the margins of geographical scholarship in order to examine the violence, predation and dispossession of the Stalin era “as material fact, as lived experience and as resonant memory” that Gregory & Pred (2007:2) remind us “erupt so vividly time and time again in our own present”. Our primary focus here is on the ‘material fact’ of the triad. This is a first step towards a deeper understanding of the role of space in the transformations in people’s lives, their imaginative universes, belief systems, and relationships with state power and of how the period of the Stalin repressions is remembered at the present time. The Stalin era five-year plans were associated with the creation of the new built environment of factories, towns, and railways, and a vast military-industrial complex, the replacement of peasant farming by giant grain factories, and the ‘socialist’ reshaping the environment, which symbolised the ‘Stalin Plan for the Transformation of Nature’ (1948). These transformations were achieved at immense human cost, the pivotal role in which was occupied by the complex, inter-related institutions of the exceptional penal monolith created during the first two decades of Soviet power and sustained thereafter.

## Methods

Historical geographic information science (HGIS) has been gaining ground in the past decade as its multiple uses for the spatial analysis of historical data have been taken up by scholars interested in past landscapes. The first stage in HGIS is database creation which, in their state-of-the-art summary, Gregory & Healy (2007) acknowledge as the most time-consuming and costly stage of any project. Scholars of the Soviet repressions face especially acute difficulties accessing data because of the censorship of relevant archive funds and a reluctance of onshore scholars to engage in research that, when published or circulated in the media, may be construed as disseminating ‘disrespect for Russia’, which has recently been criminalised. The data-deficit is particularly striking when compared with Holocaust studies, and the situation is unlikely to improve in the near future. Regional and local data down to the level of individual camps are particularly sought after by the historian of the mass repressions. Accessing data at this level depends upon local power configurations and the predispositions of individual data-gatekeepers that can sometimes work in the researcher’s favour but are extremely unpredictable. In the case-study examples that follow, we have used data accessed from national archives that opened in the 1990s and the main funds of which are now available in digital form, and from regional archives and data collected in the field and with the assistance of onshore scholars in the 2000s. 

Visualisation of the variously named violent-, lethal-, terror-, war-, or death-scapes face the universal problem that there are no solid traditions or developed methodologies in GIS-mapping that can easily be transferred for use with historical datasets. In the best-case examples, *ad hoc* teams with the appropriate range of skills are assembled for specific projects, but more usually, the task of representing data is contracted out, the resultant plethora of techniques, symbols, and legends underscoring the urgent need for the development of standard methodologies. The work of the Holocaust Geographies Collaborative is the most comprehensive and advanced among current initiatives. ^6^ The project combines archival and textual data with the spatial analytical tools of geovisualization to reveal the patterns of events as the Nazis imposed a sweeping geography of oppression across East Central Europe ( Giordano & Cole, 2020; Knowles *et al.,* 2014). The project has fixed the geolocation of 1,300 concentration camps. By layering the resultant pattern with many different types of qualitative and quantitative information in the same visual space, it has shown change in the network of camps over time"The key” as Ann Kelly Knowles explained in an interview with the National Science Foundation in 2015, “is to recognize that perpetrators and victims experienced the Holocaust at different scales, but that those scales registered--came together--in particular places at particular times …. Mapping complex data, like the development of the SS concentration camps system, inevitably shows you things you would not know--unless you make a map."( Cimons, 2015) Turning this cartographic ‘key’, we believe, is long overdue in studies of the Soviet repressions. 

The groundwork for mapping the Soviet repression is already in place. In 1998, the non-governmental organisation (NGO) *Memorial*, ^7^ that for over thirty years has been collecting testimonies, artefacts, and archival data on the repressions, published a directory, compiled by historians, N.G. Okhotin and A. B. Roginskii,of the main Soviet labour camps ( Smirnov, 1998). Together with historians and geographers from Moscow State University, *Memorial* used this directory to produce a small-scale map of the distribution of all main labour camp administrations for the period 1923–1960. ^8^ This early project served both to disseminate knowledge about the number, size and location of camps; the click of the mouse over a selected camp, accesses a panel summarizing, largely quantitative, data about the camp. The *Memorial* directory is an invaluable resource for small-scale mapping and has been used in all subsequent projects, including an online map produced by the State Historical Museum of the Gulag. ^9^ The same database was used by the present authors for their website ^10^ and by Seth Bernstein for a GIS-Soviet Repressions blog. ^11^

These uses of HGIS to represent the geography of the network of labour camps has pedagogic value, but for the victims and their descendants, mapping also has affective resonance. *Memorial* Society’s first interactive map coincided with a newly conferred right for descendants of the repressed to see their relatives’ secret files and maps like *Memorial*’s and the Gulag Museum’s helped satisfy a natural curiosity to find out more about the places where a relative was held or died. Numerous local level initiatives by civil society organisations and regional museums to emplace sites of the repression followed ( Gavrilova, 2019). The various maps produced, provide a resource for contextualising hidden family histories. It is this understanding of the importance of the placing and spacing of terror events to the act of remembering the past that underpinned Moscow *Memorial*’s project ‘ *Eto priamo zdes*’, or ‘It happened just here’. The project produces layered maps of the capital city’s ‘topography of terror’ that pinpoint the precise places in Moscow where the instruments and practices of the repression were planned and enacted by the perpetrators, and where their victims suffered ( https://www.memo.ru/ru-ru/projects/topos). An analogous project to locate the gulag’s necropolises has been pursued by Irina Flige director of the St Petersburg branch of *Memorial* and, prior to his arrest, historian, Yury Dmitriev. ^12^ These activities of emplacement are politically and geo-politically sensitive in today’s Russia. The necropolis project has been caught up in the storm of accusations and counter accusation between Finland and Russia about the perpetrators of the mass graves uncovered at Sandarmokh in Russian Karelia. Meanwhile, late 2020 saw the final act in the Russian Federation’s attempt to silence *Memorial* as state prosecutors sought to close the NGO down through the courts. This it did in December 2021, and the fate of its various projects was uncertain at the time of writing.

We have already mentioned the lack access researchers have to relevant archives. There are additionally some extremely difficult Russia-specific challenges to spatial analysis of data about the repressions. These include the doubtful reliability and interpretation of much of the available data. As an example, Nakonechnyi (2022) has shown that camp bosses falsified mortality and morbidity statistics they sent to the centre. The geocoding of spatial data, never an easy task, is made doubly difficult in Russia because of politically induced place name changes, the transitory nature of places of detention, and the intentional falsification of geolocations. Meanwhile, the generation of people who could help locate ‘where things happened’ is dying out and remains of deserted camps are, literally, sinking into the permafrost or being reclaimed by the taiga.

Qualitative data display has been employed as a pedagogic or public information tool about the Soviet repressions, but less so in scholarly work. An example of the former is an ArcGIS StoryMap internet resource entitled ‘Soviet Deportations’ that combines maps, graphs, images, quotations from deportees’ testimonies, and explanatory texts to tell the story of the peasant and ethnic deportations. ^13^ Testimonies and memoires of the victims of the repression are a major source of materials for gulag historians and there are now many thousands of published and unpublished manuscripts, video, and audio recordings available that have been collected by descendants of the repressed, human rights NGOs, museums, and filmmakers. These testimonies, some translated into English, are a rich resource that can be mined for empirical information or used for linguistic, literary and ethnographic research and to big data analysis ( Pallot, 2022). In a later section, we trace the journey of special settler through the landscape of repression using his short memoire to demonstrate that mobility was integral to the experience of exile. For some, this was the terror of the *etap* or transportation that delivered people in Stolypin carriages, analogous to the Holocaust cattle trucks, to camps and exile ( Pallot, 2015a). For others, as the example we use below illustrates, it was the perpetual motion of being relocated from construction site to construction site, as the resource frontier pushed ever outwards. 

In this article, our aim is to present examples of how different quantitative and qualitative data sources can be mapped to visualize processes that cannot be captured easily in a print format including the relationships between the diverse elements of the terror landscape and between the stasis and mobility of the ‘ *zek* people.’ We do this for three spatial scales at each of which we use the techniques offered by HGIS to show how mapping can give greater clarity to the boundary questions to which we draw attention above. Mapping can also help to dispel some of the popular misconceptions about the landscapes of repression that continue to have currency at the level of public discourse, inside and outside Russia.

## The temporal and spatial unfolding of the gulag at the national level

In this section, we use the directory that Okhotin and Roginskii compiled for *Memorial* to challenge popular myths circulating about the spatiality of labour camps. ^14^ The sort of myths we have in mind range from the casual cliché that offenders in Russia were (and still are) ‘sent to Siberia’, to misconceptions about camps’ presence in the landscape. These have been reinforced by the maps that typically are included in the general histories of the gulag which constitute the main reference point for non-specialists interested in ‘the camp’ and repressive landscapes (see, for example, the maps in Applebaum, 2003:120–21; Figes, 2007: xiv; Khlevniuk, 2004). Susan Grunwald (2022), in her use of historical GIS, has already dispelled the assumption that German prisoner-of-war camps mirrored the location of the main gulag camps. Our aim for the small-scale mapping using the Memorial directory is to convey how the gulag unfolded in space and time across the vast expanse of the unevenly populated Soviet landmass. We were able to do this by geolocating each main administration of the over 400 labour camps listed in the Directory and the demographic statistics recorded for them. As we describe below the demographic data was not systematically collected and recorded by the centre, so the time-series is incomplete.

**Figure 1.**
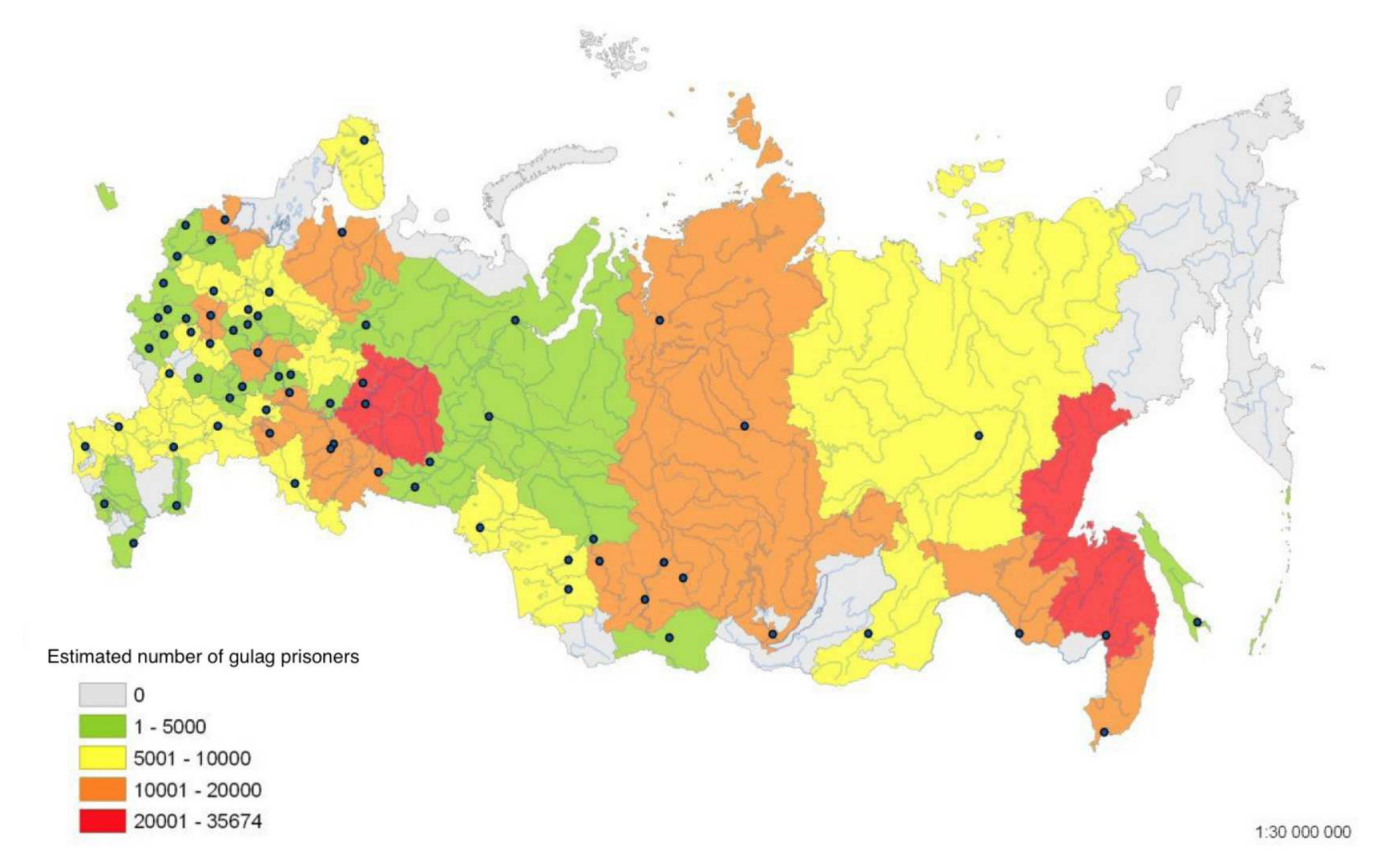
The regional prison population in the main administrations of camps in the GULAG for one average year between 1931 and 1941 (Data source: Smirnov, 1998. *Sistema ispravitel'no-trudovykh lagerei v SSSR: Spravochnik* (The system of correctional labour camps in the USSR: Directory), NGO Memorial.

The typical visual representation of the chief labour camp administrations on point maps conceals the fact that camps were strongly differentiated in their spatio-organisational structure, contained widely different numbers of convicts, and they existed for variable lengths of time. Camps, like those in Moscow city, might occupy a relatively small area in a metropolitan suburb or, by contrast, they could consist of a network of facilities covering hundreds of square kilometres in the *taiga*, as was the case with the camps in the GULAG forest directorate. The prisoner population is one measure of a camp’s dominance in the landscape. For our website ^15^ we produced a map series of the average prisoner population in each major geographical region across the USSR for ten-year periods. The choice of the ten-year envelope was dictated by the fact that data in the *Memorial* directory is a compilation of one-off censuses for specific camps in different years, and not a complete time-series for the convict population in all camps. The taking of single year 'snapshots' therefore risked vastly underestimating prisoner numbers. We tackled this problem by treating the location of camps and prisoner numbers as two separate datasets and combining them to produce a set from which we estimated the average number of prisoners per year in each camp for the period’s duration. For example, if there were figures for a particular camp every year for a five-year period, we would sum and divide by five taking account the length of the camp’s existence. If there were figures for only one year, we would take that one. If there were two years, summed and divided by two and so on. We then summed the average for each camp for a ten-year period to achieve an overall total for that decade. This gave us an approximate guide to the geographical distribution of prisoners across the gulag for successive ten-year periods. In Figure 1, we give an example of the prisoner population map for the Russian republic (RSFSR) in 1931–1941. This period encompasses the pre-war expansion of the first Soviet concentration camp outwards from the Solovetsky islands in the White Sea and the proliferation of camps associated with the 1937–38 Great Terror.

No less problematic than capturing the differentiation in the population size of camps is their temporal fluidity. Camps were created for the fulfilment of specific building or resource extraction tasks and were dissolved when the task was completed. Large camp complexes - those with the prisoner population exceeding 50,000 for the period of their existence – were not confined to the area east of the Urals, but first appeared in the European part of the country ( Averkieva, 2015; Averkieva, 2016). The complexes included both those that were ephemeral and those that were long-lived. Among the former were the notorious camps of the 1930s - BelBaltLag and Dmitlag - founded to build canals in European Russia and the enormous Bezymenlag in the Ural-Volga region that lasted just six years (1940–6). Among the longer-lived camps were the mining camps in Arctic Siberia – Noril’lag and Kolyma - and Karlag, the latter covering an extraordinary 6,800 kms ^2^, in the dry steppe of Kazakhstan and longer lived than any other gulag camp. The representation of the *Memorial* data for the whole of the gulag’s existence in a single map, typical for the mainstream gulag histories, not only fails to capture its mobility but also creates the impression that to traverse the landscape anywhere in Russia between 1930 and 1960 was inevitably to encounter a camp. 

**Figure 2.**
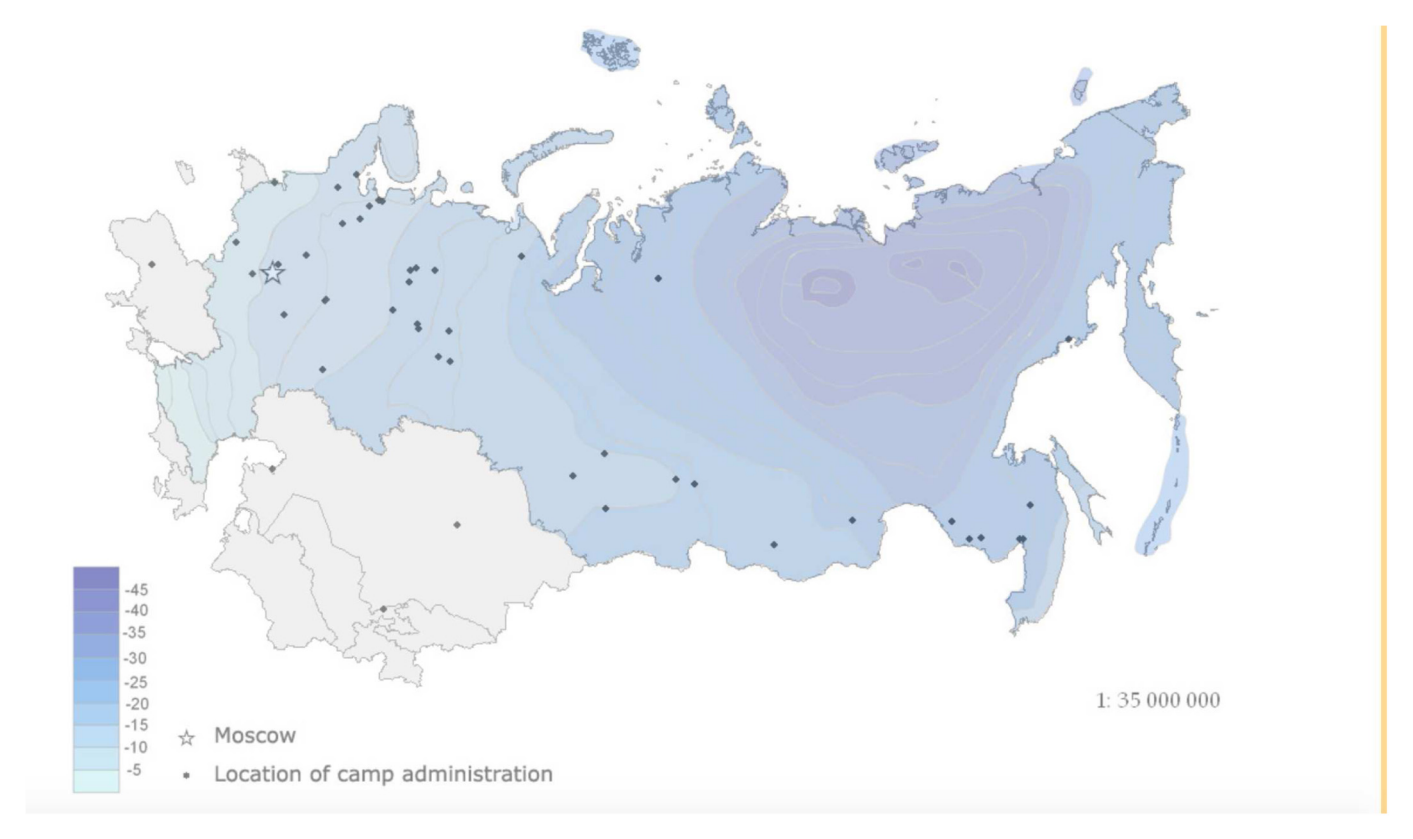
Distribution of main labour camp administrations 1937–1938 with overlay of January average temperature in degrees centigrade. (Data source: Smirnov, 1998. *Sistema ispravitel'no-trudovykh lagerei v SSSR: Spravochnik* (The system of correctional labour camps in the USSR: Directory), NGO Memorial.

**Figure 3.**
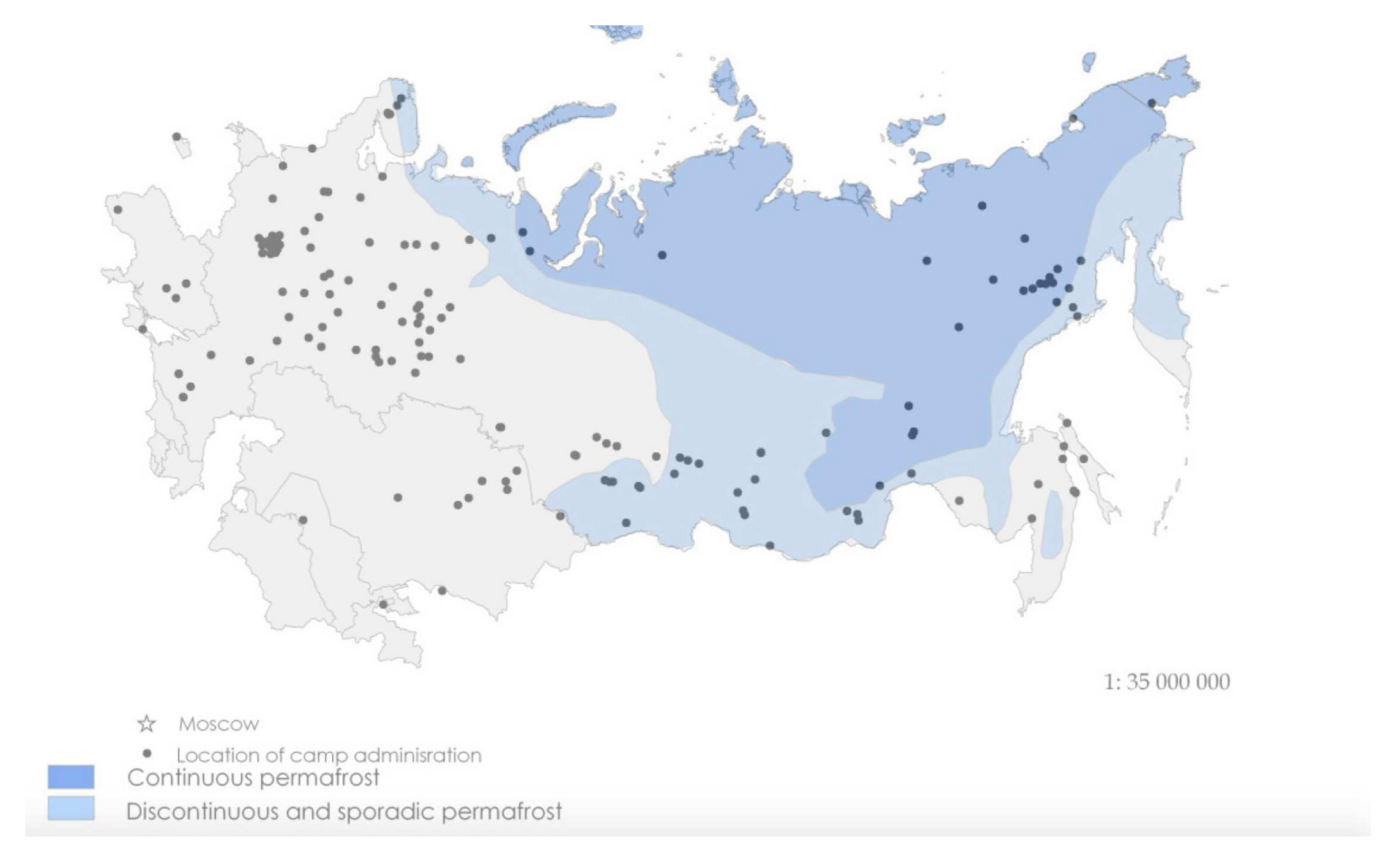
Distribution of Main Labour Camp Administrations, 1953 and 1954 with overlay of extent of continuous and discontinuous permafrost (Data source: Smirnov, 1998. *Sistema ispravitel'no-trudovykh lagerei v SSSR: Spravochnik* (The system of correctional labour camps in the USSR: Directory), NGO Memorial.

 In our website, we used the data in the *Memorial* directory of the location of each camp’s headquarters and the date of their foundation and closure to construct a map series of the main camp administrations for two-year periods relating to the gulag’s rollout from 1929 to 1960. All the maps can be overlaid with average January temperatures and the extent of continuous and discontinuous permafrost. Figure 2 and Figure 3 are distribution maps of camps in 1937 –1938 and 1953–54 from this time series. They show that the spread of camps into the most environmentally extreme regions post-dated the years of the Great Terror. In the latter years of the 1950s, the gulag contracted inwards to the inner periphery to form an arc in the European Russian north and Urals where the majority of penal facilities has remained to the present time.

The large Siberian camp complexes came into being against the backdrop of an expansion of camps west of the Urals and largely avoiding the non-Slavic republics. While each individual map from the time-series tells a different story about the gulag explicable by changing domestic and external events, when the time-series is animated, the story is of the expansion into the geographic peripheries followed by hollowing out and contraction. ^16^

## The material landscape of repression at the regional level

At regional and local scales, the data that can be drawn upon are more fragmented than at the national scale. The fundamental background data on locations, dates, and the socio-economic and criminogenic characteristics of the people who inhabited the landscapes of repression remain firmly out of reach of the researcher in the OGPU/NKVD/KGB and Prison Service archives. There are some regions where civil society organisations, local museums and ‘individual enthusiasts’ have put together collections of materials to tell the story of their region. In the next two sections, we use such materials from the Perm’ region in the Urals, to highlight the spatiality of the production of the material landscape of repression in one region. 

Perm’, or Molotov oblast as it was known 1940–57, lies on the western flanks of the Urals range and is an old industrial region based on iron ore, salt, and hydrocarbon resources dating from the 18 ^th^ century. The northern part of the region is an area of marsh, acidic soils, and extensive boreal forests and it was the latter’s suitability for the USSR’s developing paper and cellulose industry and peripheral location that led to it being chosen as a destination for the ‘special contingent’ of convicts and exiles from other parts of the USSR. The transfers began in the earliest years of the mass repressions in 1929–30 and continued through to the dismantling of the gulag as an administrative entity after Stalin’s death. Perm’ region was not one of the most notorious gulag sites - a maximum 45–46,000 prisoners, modest by the standards of the time, were held in the various camps in the north of the region after WWII. But Perm’ was among the regions with especially large numbers of deportees - 90,000 in 1950s ( Polyan, 2001; Suslov, 2000).

These latter had arrived in two main waves consisting of kulak families exiled from Ukraine during the collectivisation drive, and ‘suspect’ ethnic groups (Volga Germans, Crimean Tatars, Balts, and other minorities) and Nazi collaborators (largely Ukrainian OUNites and Vlasovites) deported from western regions after 1941. ^17^ Other categories of new arrivals in northern Perm’ were members of the volunteer labour army ( *trudarmiya*) consisting of ethnic German Russians, Soviet soldiers released from German POW camps who were confined in four filtration camps in the oblast, and German, Italian, Hungarian, and Austrian prisoners-of-war, of which there were approximately 25,000 in the whole region. The new arrivals could be assigned to *spetsposelenii* (special settlements) or to labour camps, under the appropriate main administrations (GULAG and GUVPI) within the Interior Commissariat.

The insertion of hundreds of thousands of the special contingent, along with the military detachments of guards and NKVD officers responsible for their ‘re-education’ and mobilization to fulfil plans for mining and timber harvesting in an otherwise sparsely populated area, inevitably had an impact on the material landscape and demographic and ethnic composition of local populations. The transformations of the material landscape consisted of reshaping the density and pattern of human settlement, and the development of industrial plants, building the socialist new town of Berezniki and the region’s transport infrastructure. There was an ecological impact as well, with logging leading to deforestation and changes in river biomes because of ‘log driving’ and river dredging for diamonds. Where population was concerned, the number of special settlers who arrived in the four most distant rural districts of the oblast’ during the Stalin period expanded their existing populations by between 52 and 156 percent, and by 77 per cent in the Komi-Permyak autonomous oblast’ ( Pallot, 2002:1065). 

Here, we focus on how the repression changed the region’s settlement geography. The number of prisoners and special settlers in Perm’ region reached its height in the post-war years. In 1952, gulag camps in the Perm’ region were located at the northern end of a continuous arc extending south into neighbouring Sverdlovsk and on into Chelyabinsk regions. Within this arc, there were overlapping hierarchies of habitation sites produced by the camps. These could be a cluster of buildings that existed for the camp’s duration and may have turned into permanent settlements, and others that were temporary and little more than a collection of dugouts or barracks. The camp administration, or *shtab*, was the most permanent of the structures and, typically, it was located in a pre-existing settlement. Below the *shtab* in the organisational and spatial hierarchy were camp branches ( *otdeleniya lagerya*) in which the majority of prisoners were accommodated. These could also be set up in existing settlements. In 1952–3, the most northerly camp in Perm’ oblast, Nyroblag, had a population of 25,113 prisoners and multiple sub-divisions, which contrasted with the much smaller Molotov camp founded to build an oil refinery in the regional capital that had under 5,000 prisoners and existed for three years, 1950–3.

The lower levels in the hierarchy of places making up camps in this peripheral, forested region were ‘camp points’ ( *lagpunkti*). Below these in the hierarchy were separate camp points ( *otdel’nye lagernye punkti*) and the ephemeral, ‘companies’ ( *komandirovki*), that might exist only for a few months to clear a particular area of forest for example, and the ‘special assignments’ ( *spetsuchastki*) provisional small-scale units serving similar purposes to the *komandirovki*. The ability to map at this level depends upon the preservation of location data in the archives of regional government and *kraevedcheskie* museums, local informants, and remote sensing. We were fortunate that Perm’ region had an active branch of the NGO *Memorial* and was prepared to collaborate with us on a mapping project. From the late 1990s and early 2000s, historians in the *Perm Memorial* had begun assembling materials on the repression in the region and publishing the results. These materials provided us with the raw data we needed, which consisted of the name of the command posts in the north of the oblast, the settlements subordinated to them and the distance of each from the command post, to create maps showing the distribution of special settlements. These and other data are now available on-line on the Perm’ Memorial website. ^18^ Additionally, we benefitted from lucky finds in the central state archives, including original maps of the most northly camps in the oblast.

The figure below shows the complex spatial hierarchy of Usol’lag in northern Perm’ oblast, consisting of three sub-divisions each with its own subordinate hierarchy of places.

The original was a 1:375 000 scale map of Usol’lag dated 1945 and entitled ‘Map of the Operational Territory of Ussollag-NKVD’ ( *‘Karta rayonov raboty Ussollaga NKVD’*). ^19^ In addition to the sites where prisoners were confined, it shows topographic features, rivers, roads, and forest paths. It also has some places marked in red pencil, which we surmise to be projected locations for new *lagpunkti*. The camp headquarters was in Solikamsk and the whole camp occupied an area of about 60–70,000 kms ^2^. Usol’lag consisted in 1945 of three camp branches (Ust-Yazva, Nyrob, which later was to become a separate camp, and Bondug), 21 camp points, and fifty-four special assignments. There is a second, schematic, map at a larger scale in the archive of one of the camp’s branches that shows the lines of communication – rivers, roads, forest paths - between headquarters of Nyrob camp branch and its network of subordinate sites. The distance to each is given in kilometres, with the most distant special assignment located 90 kms from the headquarters. Georeferencing the data was tricky because camp points and special assignments were in virgin forest. The majority of these places have subsequently been claimed by the *taiga* but some have remained.

 In Figure 5, we have added a shapefile of current settlements to the map we created of the Usol’lag and applied methods of spatial analysis to identify which places were founded by the camp, which disappeared, and which still exist today. We did this by creating a buffer zone around each site to take account of possible author inaccuracies in the original mapmakers’ placing the dots and then, 'intersecting' them to leave only those villages located within the buffer zones. Finally, we grouped the settlements by the date of their foundation.. Eleven of Perm’ region’s current thirty-three penal facilities are in the former territory of Usol’lag, occupying the same sites, and in some cases, the same buildings as during the 1950s, which speaks to the continuity of carceral landscapes in Russia ( Moran *et al.,* 2011; Pallot, 2005). ^20^. We were able to confirm their location and function in a field trip to the region in 2016. The map illustrates the urban or region-forming ( *gradoobrazuyushchii*/ *regionoobrazyushchii)* role of the gulag. 

Successive waves of exiles and deportees are the other part of the story of settlement change in Perm’ region in 1930–53. The settlements occupied by ‘special settlers’ were the lowest rung in the settlement hierarchy of the repression landscape. The list of command centres and settlements that made up the special settlement network in Perm’ was compiled by a researcher from *Memorial* on our behalf from three regional archives, and from GARF, Gosudarstvennyi Arkhiv Rossiiskoi Federatisii), the state archive of the Russian Federation. ^21^ The total number of special settlements created in the oblast was 1,700 which were linked to one of 316 command centres or *komandatori*. We produced a series of location maps from the archival data using the names of command posts and the special settlements (where given) subordinated to each. In producing the map series, we created georeferenced datasets of command points and special settlements that were linked to a variety of parameters in which we were interested. These included settlement longevities, the size of the special contingent on arrival, and at intervals thereafter. We had to overcome the familiar problems of georeferencing settlements from this era. Commandant No. 49, for example, simply recorded the ‘contingent’ as residing in 10 places between five to 50 kilometres (kms) from the command point in Kungur. To overcome this problem, we used a shape file in QGIS to produce a map of the radius within which special settlements subordinate to the oblast’s command posts were located. Figure 6 shows that while the network of special settlements subordinated to any one command point was usually dispersed, the jurisdictions of the *kommandatori* overlapped.

**Figure 4.**
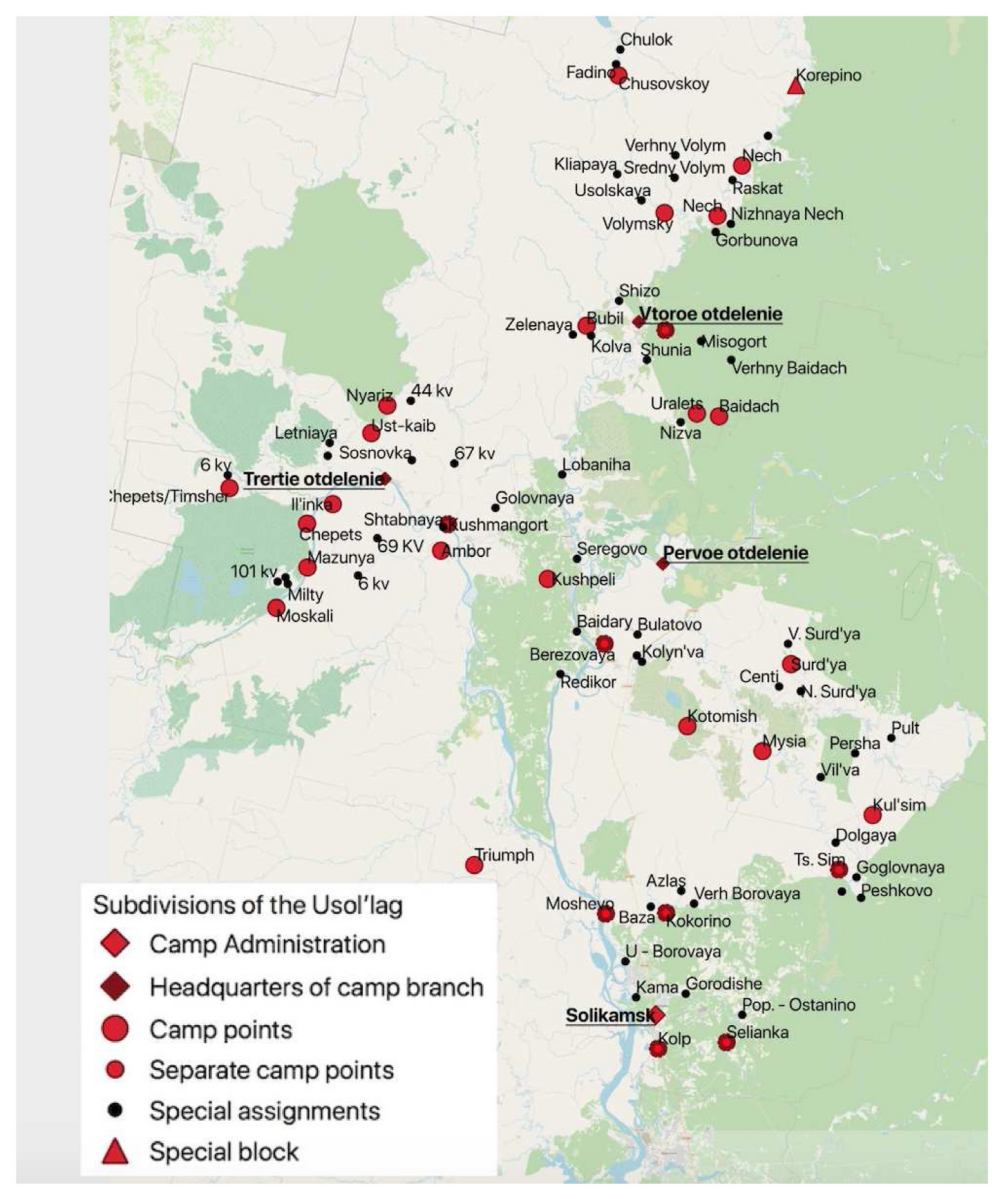
The hierarchy of permanent and temporary places of confinement in Usol’lag, Northern Perm’ (Molotov) Oblast (Map constructed from the original in GARF: f. 8131, opis 37, delo 2481).

**Figure 5.**
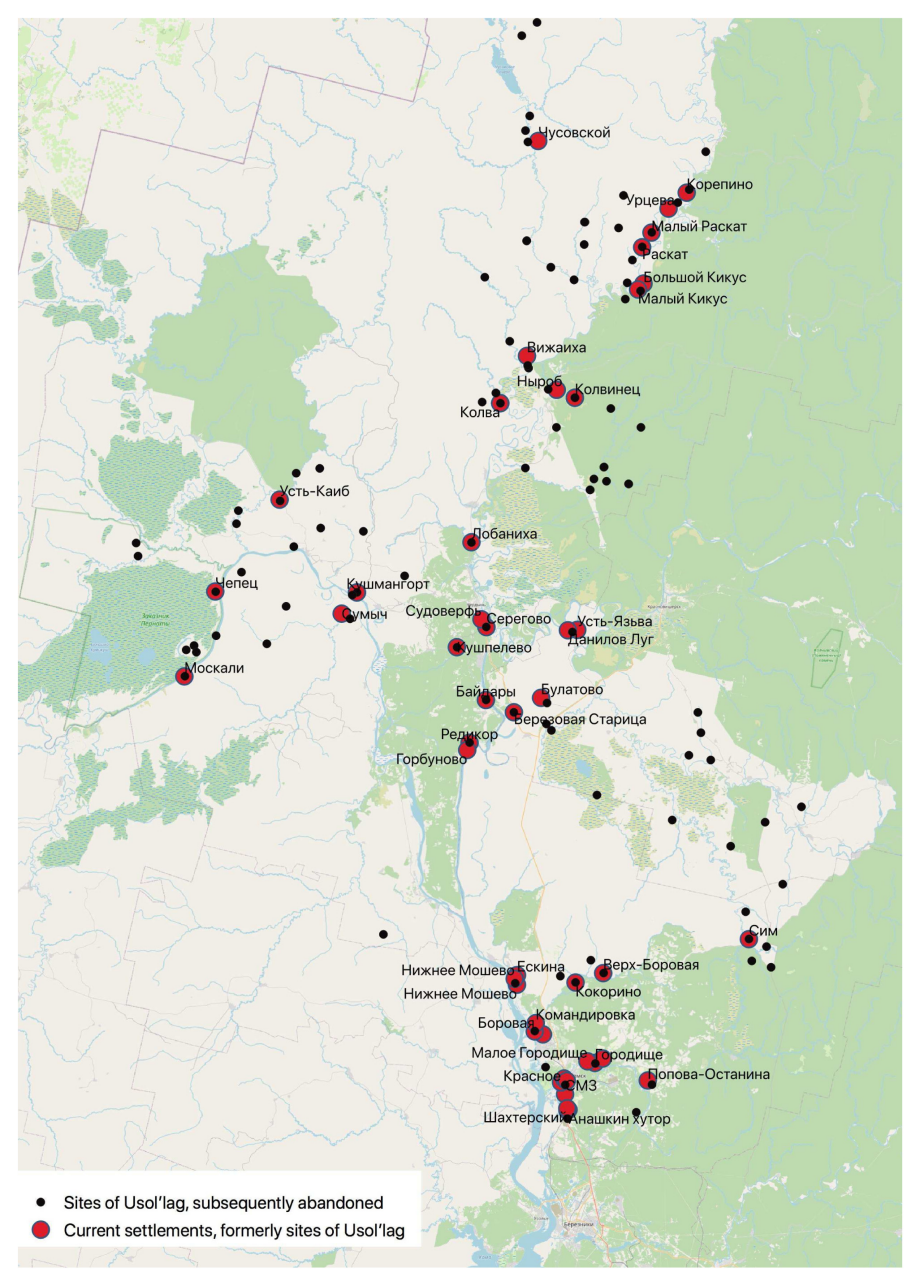
Surviving and disappeared sites of special settlements ( *spetsposelenii*) and GULAG settlement points in the eastern part of the territory the former Usol’lag, northern Perm’ krai, 2020. (Constructed using the data in Figure 4, Spisok Naselennykh Mest Permskoi Gubernii,Permskoe Gubernskoe Zemstvo, 1904 and google map of Perm’ oblast).

**Figure 6.**
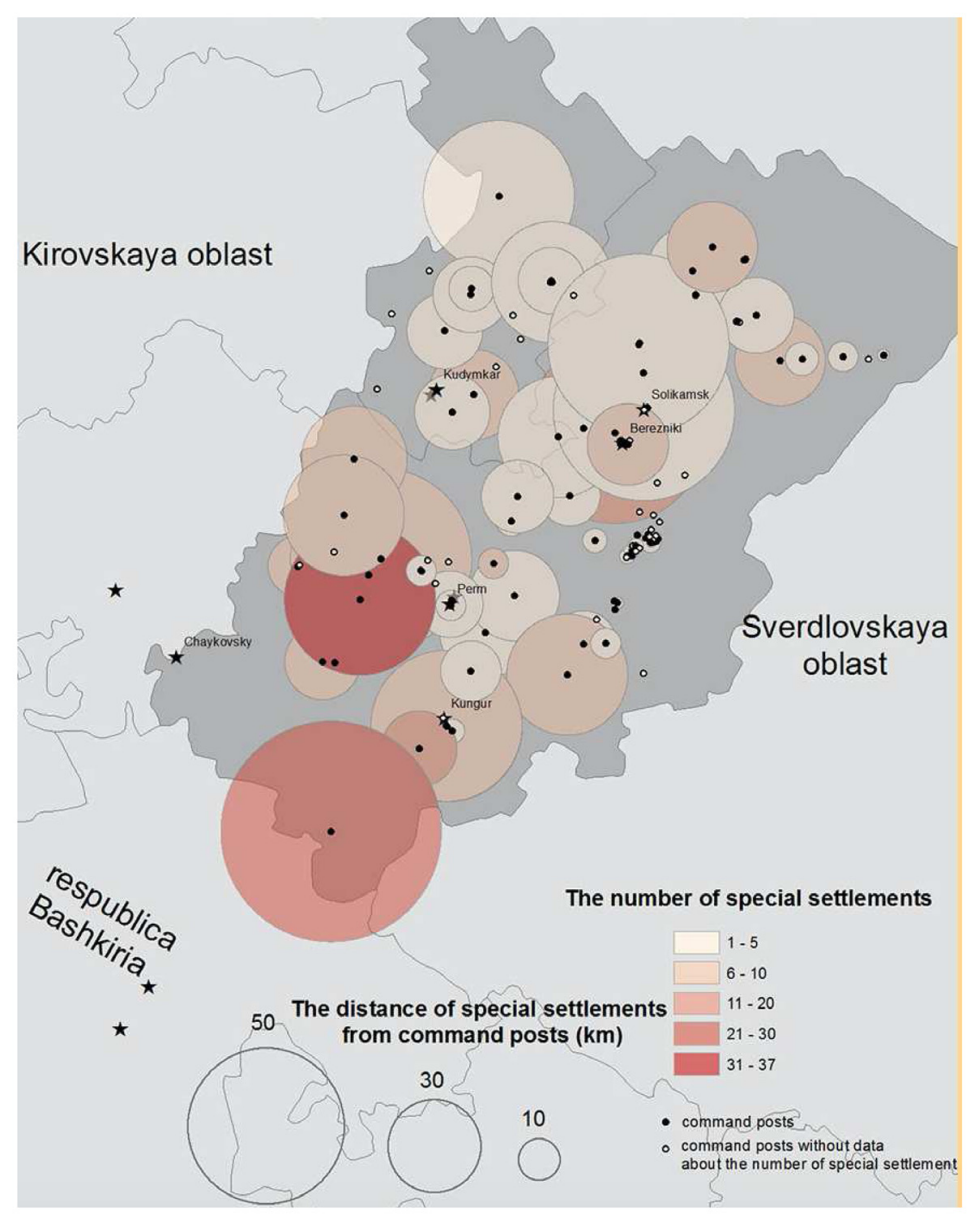
The number of special settlements and their distance from the command posts to which they were subordinated in Perm’ (Molotov) oblast, 1952. (Sources: Arkhiv ITs GUBD, fond 21, opis 1, delo 1 and 6; PermGANI, fond 105, opis 18, delo 195; GARF fond 9479, opis 1, delo 496.).

**Figure 7.**
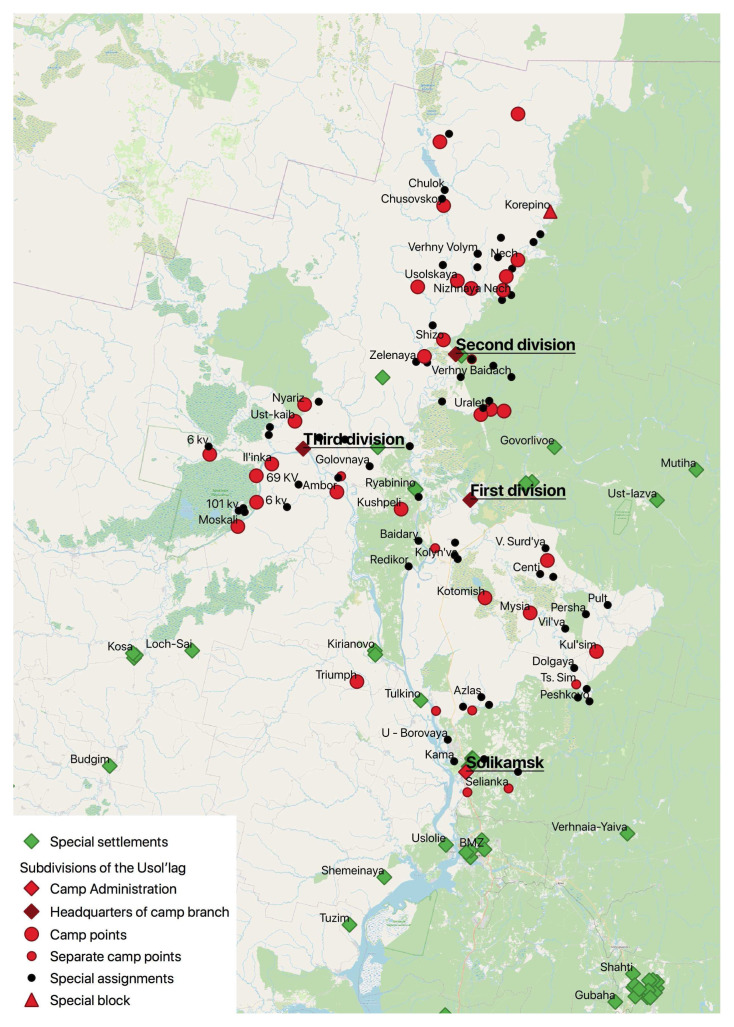
Integrated map of gulag and special settlements in the territory of Usol’lag 1945–50.

In Figure 7, we have used the databases we created for the location of gulag settlement hierarchies and special settlements in Figure 4, Figure 5 and Figure 6, to represent visually the spaces occupied by the two different types of carceral institutions. As we have already observed, these are typically treated separately in history texts, but their joint visualization conveys the intensity of the penetration of the northern forest by the apparatus of repression. The resultant map dispels the idea that labour camps and special settlements operated in discrete spaces, indicating that a detailed exploring the day-to-day relationship between the two is in order. At the same time, the location of some special settlements in the margins of an already marginal region raises interesting questions about the hierarchies of ‘detriment’ experienced by different categories of the repressions’ victims.

## An individual’s journey through the repression landscape

**Figure 8.**
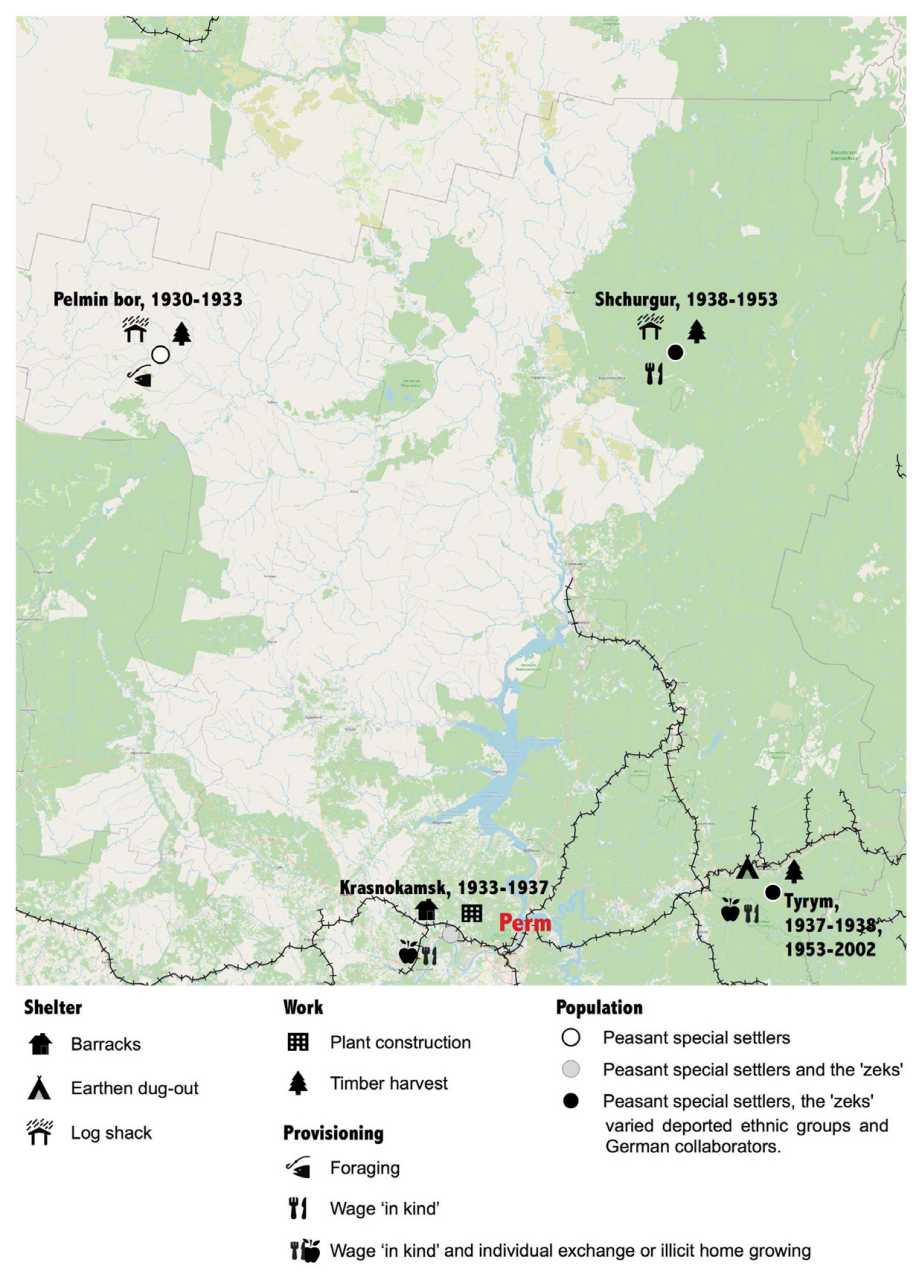
Filip’s journey. (source: see footnote 22)

 Following the example of Holocaust Project’s mapping of individual geographies, we follow the journey of an anonymised deported peasant through the new settlement landscape we have described above. We shall call him Filip. Filip was deported along with his family from Western Ukraine in the mass deportations of kulaks in 1929–1931. ^22^ We were interested to see whether the geovisualisation of Filip’s journey would reveal aspects of his story that that might missed reading written text. In Figure 8, we make a very simple map to show the places to which Filip Ipatevich was sent to work as a special settler, and at each site we have added symbols representing his main food source, his living accommodation, his work, and the people he lived alongside. What surfaces in the mapping of these four simple variables is a polychromatic story of the settler experience, as it unfolded through space and time, all sourced from just seven pages of field notes as Filip told his life story.

Filip’s journey began in 1930 in a peasant village in Vinitsa in Western Ukraine, when he was eight years old. His father was deported as a kulak, or rich peasant, and, by definition, class enemy in Stalin’s tripartite classification of peasantry. The 2,500 km journey by train, foot, and barge to the Komi-Permyak region in the north Urals brought Filip, his five sisters, and parents to Gainy on a tributary of the Kama River from which the family was delivered 75 kms upstream to an uninhabited place in the *taiga* to which the name Pel’min Bor had been given. In Filip’s words this was a place that “nobody would ever be able to find, had no land for growing, and was freezing cold” ^18^. Emaciated and ill, the peasants delivered to this place could not fulfil their timber harvesting quotas, the requirement for deliveries of food, but, in any case, these were the famine years of 1932–3 and it is unlikely that starvation would have been avoided. Having eaten the horses supposed to be dragging timber to the riverbank, in the winter the settlers were reduced to eating bark and reindeer moss. They suffered from scurvy and ‘died like flies’. In 1933 Filip’s family were transported south to another special settlement, Krasnokamsk, the location of a projected Paper and Cellulose Plant, destined to be the largest in Europe. Here Filip encountered a larger and ethnically more varied population of forced labourers. His father worked as an iron monger and Filip, now 11-years, was put to work carting bricks to the building site. For the first time in three years, Filip had bread to eat, which his parents were able to exchange for small household items his father risked making on the side.

The completion of the Krasnokamsk Paper and Cellulose Combine in 1937 saw Filip’s family relocated again, this time to a mining region on the west facing slopes of the Urals. The family was fortunate to have been moved before the Great Terror of 1937–8 which claimed many special settlers at the combine, who were executed for spying and anti-Soviet agitation. This time, the destination was Ust’-Tyrym, but Filip was immediately sent to Shurgur, a more northerly special settlement. This was a period of extensive prospecting for diamonds in the Urals and the Filip’s new home was one of the sites developed by Uralmaz, the diamond enterprise. The task of dragging the rivers was given to the work army ‘volunteers’, special settlers and gulag prisoners. However, Filip was almost immediately redirected to harvest timber on the Vishera river further to the north to supply the newly opened Vishera cellulose and paper combine ( Pallot, 2002). Clear-cutting of pine stands, free floatation of logs downstream ( *splav*), and the excavation of rivers effected major changes in the river basins and forest ecological in this part of the Urals, which is still visible in the landscape today. The camps and special settlements in the northern taiga were among the deadliest of the gulag and especially dangerous was the job of straightening fast moving logs with long staves as they raced past the crib piers ( *ryazhi*) constructed in middle of the rivers. Filip had to make his way onto the pier by jumping across the floating logs and was lucky to survive. After Stalin’s death, he re-joined his family in Ust’-Turym, from where he witnessed the sequential emptying of the special settlements of different national groups that had made up their population. When interviewed, Filip, now widowed and his children long gone, was living on his state pension, plus a supplement he received as a victim of the repression in the largely abandoned, isolated settlement.

The landscapes produced in the years of Soviet repression were the product of hundreds of thousands of journeys like Filip’s. Putting together the puzzle of personal journeys through the landscape of the repression, is a valuable complement to the available quantitative data and provides a path into a more experiential writing of the Soviet repressions’ spatiality. As Sarah Young (2022) has observed there are now hundreds of ‘gulag testimonies’ available in digitised form available for analysis, most running into the considerably more pages than the few pages in a field notebook that Filip’s interview occupies. These testimonies are an enormously rich source for using HGIS to visualise the journeys that individuals were forced to undertake during the Stalinist repressions.

## Conclusion

Our understanding of repression in this article is the use by a state entity of violence against its citizenry that violates their rights and is used in pursuit of military, economic and/or political objectives ( deMeritt, 2016). This describes the Stalinist repressions. Our focus has been on how this regime of punishment transformed the material landscape as an expression of the coercive power of the agents of repression working through the labour power of their victims. The Stalin era five-year plans were associated with the creation of a new built environment of factories, towns, and railways, and a vast military-industrial complex, the replacement of peasant farming by giant grain factories, and the ‘socialist’ reshaping the environment, symbolising the ‘Stalin Plan for the Transformation of Nature’ and ‘Communist conquest of the Arctic’. These transformations were achieved at immense human cost, a pivotal role in which was occupied by the complex, inter-related institutions of the exceptional penal monolith created during the first two decades of Soviet power and sustained thereafter. 

Our intention has been to bring to the attention of geographers interested in violent and lethal geographies the rich potential there is for including the history of Soviet mass repressions in their enquiry. It is perplexing that geographers pay so little attention to the former Soviet Union, given their embrace of the theorizations of Foucault and Agamben. In the past, politics and ideology, as well as almost insurmountable practical obstacles, inhibited research into the Stalinist repressions, but these obstacles are more muted today. Meanwhile, the rise of right-wing populism and what Haney (2016) describes as ‘penal nationalisms’ in the ‘global east’, underlines the pressing need to excavate the dark underbelly of Soviet communism, so that its legacies are properly understood. For historians, our message is that visualisation of the data they collect in archives can open new avenues for enquiry and challenge the boundary assumptions that flow from too uncritical adherence to Soviet era taxonomies. The new generation of historians are engaging with the question of the spatiality of the repressions, but they have yet to embrace the new methodologies for their representation. The examples of cartographic visualisation of data that we have presented also challenge popular tropes about the Soviet repressions and argue for care to be exercised in drawing comparisons of the USSR’s carcerality with conditions in camps and places of exile, elsewhere. We hope that we have made a start in conveying the extraordinary complexity of the material landscape of the Soviet repressions, paving the way for discussion of the impact on the lived experience of the people who inhabited it and of how it is remembered today.

## Data availability

Interview data used in this study is currently under embargo as part of the GULAGECHOES project (ERC No. 788448) in Helsinki University. These materials contain the personal data of vulnerable subjects and are under embargo until the end of the project in April 2024. Once the project ends, the data will be reviewed by the Ethics Advisory Board of the GULAGECHOES project and Helsinki University Ethics Review Committee to determine which interviews will continue to be embargoed and which will be deposited in a Zenodo repository as data. This is in line with the instruction of the ERCEA which approved the project Data Management Plan, signed by Vera Lothman 6/03/2019 Data Protection Officer of Helsinki University.

The underlying data used to construct Figure 8 cannot be shared because they are included in the materials that are being gathered for the GULAGECHOES project (ERC No. 788448) as outlined above. For data queries please contact the corresponding author at judith.pallot@helsinki.fi.

## Footnotes

^1^ This quotation is taken from the online version of the original Thomas Whitney translation that can be found at https://royallib.com/read/Solzhenitsyn_Aleksandr/the_gulag_archipelago_volume_1_an_experiment_in_literary_investigation.html#0

^2^ Nils Christie’s ground-breaking text on prison privatization in the twentieth century uses the gulag analogy in its sub-title: N. Christie, N., *Crime control as industry: Towards gulags, western style*. Oxfordshire, 1961,

^3^ Solzhenitsyn’s legacy is extremely complicated, not least by his embrace of right-wing nationalism, support of President Putin, anti-Semitism in Gulag Archipelago, and general railing against ‘western’ liberalism. For a short review of the controversies that swirl around him, see the piece by Cathy Young, *in Quillette* for 2018: https://quillette.com/2018/12/21/solzhenitsyn-the-fall-of-a-prophet/legacy last accessed 20/08/2020

^4^ The 101st kilometre rule, refers to the prohibition people expelled from Moscow, Leningrad, and other large metropolitan centres to live within 101 kilometres from them. The restriction was largely imposed on anti-Soviet offenders, but the term passed into more general use in the USSR to refer to various ‘undesirable elements’ who periodically were removed from large cities to the end of the USSR.

^5^ The OGPU was the secret police of the USSR or Joint State Political Directorate. It was superseded by the NKVD, which was the People’s Commissariat for Internal Affairs, which in turn was superseded by the MVD, or Ministry of Internal Affairs.

^6^ This is a project originally funded 2008–2012 by the National Science Foundation involving the collaboration of a team of historians and geographers. Some of the materials are already available on the site of the Standford Spatial History Project | Center for Spatial and Textual Analysis ( https://cesta.stanford.edu/research-projects/core-research-groups/spatial-history-project) (stanford.edu) at http://web.stanford.edu/group/spatialhistory/cgi-bin/site/project.php?id=1015. Some are also available in the Holocaust Museum in Washington DC.

^7^ Memorial website: https://www.memo.ru/en-us/

^8^ https://museum.memo.ru/camp_map/

^9^ Available from https://gulagmap.ru/.

^10^ Author website: http://www.gulagmaps.org.

^11^ Available at ( http://www.abstractualized.com/2015/10/mapping-gulag-over-time.html).

^12^A map of the mass graves so far discovered and pictures are available at https://en.mapofmemory.org and in the collections of the virtual museum of the gulag, which is accessed at www.gulagmuseum.org/start.do?/ Irina Flige continues her research despite the liquidation of *Memorial* The Karelian historian Yury Dmitriev, is serving a 15-year prison sentence (For details see Rights in Russia blog of Anna Yarovaya: "The Dmitriev Affair" )

^13^ https://www.arcgis.com/apps/Cascade/index.html?appid=5196c3c6897d4fe7a21b1293f9b004a2 The editor/creator of the resource is Chelsea Smith-Antonides of University of Oklahoma Digital Library Services.

^14^ The Directory is available in both printed ( Smirnov, 1998) and on-line form at old.memo.ru/history/nkvd/gulag/. The data are presented in the directory in four parts each dealing with camps subordinated to different central, regional and functional directorates of the GULAG. For each named camp the following are given: official names, date of foundation and closure, postal address, place, prisoner population for all the years for which records were found, the governor(s), location of the camps archive, archive source. The on-line version of the directory can be found by entering Система исправительно-трудовых лагерей в СССР (memo.ru) into a search engine or going to the main memorial site at https://www.memo.ru/en-us/ and following the links to Collection and Data Bases, and then the drop down to “statistics and chronology” (the latter, only in Russian).

^15^ Author website: www.gulagmaps.org

^16^ Different animations of the location changes of camp administrations and populations can be found at http://www.gulagmaps.org/maps/map.php?series=1&map=2960; Seth Berstein’s video of the point maps can be viewed on youtube at: https://www.youtube.com/watch?time_continue=4&v=XpXPN3PS1oE&feature=emb_logo and https://www.youtube.com/watch?time_continue=86&v=55VgTowioFI&feature=emb_logo.

^17^ OUNites were members of a Ukrainian insurgent army formed during WWII that aimed to create a German-aligned nation state and the Vlasovites were USSR citizens who joined the Russian Liberation Army to fight alongside the Germans against the USSR under former Red Army general A. A. Vlasov.

^18^ Perm’ Memorial website: http://pmem.ru/index.php?id=4

^19^ GARF: f. 8131, opis 37, delo 2481. The map was found in a file of a Prosecutorial custodial supervision report from 1945 -1946. The document case consists of 341 sheets, mainly consisting of correspondence between the prosecutors’ headquarters in Moscow and regional authorities. They do not contain data about the number of the inmates in different camp points, or who these inmates were. We are grateful to Mikhail Nakonechnyi, for allowing us to use the copy of the map that is in his personal possession.

^20^ Subsequently, the most distance colonies, including those in Nyrob, have been scheduled for closure because of cost and falling prisoner numbers.

^21^ The local archives were ITsGU MVD po Permskomy krayu (the Information Centre of the Main Administration of the Ministry of Internal Affairs of the Perm’ Territory), Permskii Gosudarstvennyi Arkhiv Noveishei Istorii (PermGANI - the Perm State Archive of Recent History), and Gosudarstvennyi Arkhiv Rossiiskoi Federatisii (GARF), the state archive of the Russian Federation. Perm’ *Memorial* subsequently developed an interactive map of all the special settlements in the territory. For each settlement, the data recorded in the archive is accessed by a link. These include the date of origin, population data for different years, population composition by age and ethnicity where available, and when the settlement ceased to exist or still exists today. These data are available on-line at http://pmem.ru/index.php?id=4.

^22^ The interview was taken during field work in Northern Perm’. All the interviews taken for the gulagechoes project in Russia are anonymised. The interview was recorded in a field notebook, so it is not *verbatim*. The typed-up filed notebook extract is currently stored in the private shared space in Helsinki University with other sensitive data collected for the GULAGECHOES project. Along with all interviews taken for the project, this interview is embargoed until the end of the gulagechoes project, at which point a decision will be made whether, when, and under what conditions to place it in an open access archive. Additional information is available in the data availability statement.
