# Supplementary material for: Mapping the landscapes of the Stalinist mass repressions
Source: Open Res Eur. 2022 Apr 7;2:44. [Version 1] doi: 10.12688/openreseurope.14410.1 (PMC10446028; doi:10.12688/openreseurope.14410.1)
Supplement: Peer review history

# Peer review: Reviewer response for version 1

**DOI:** 10.21956/openreseurope.15554.r29032

**Published:** 2022-04-28

**Authors:** Gavin Slade

**Affiliations:** 1 School of Sciences and Humanities, Nazarbayev University, Nur-Sultan, Kazakhstan

This is a really useful piece of writing that helps immensely to frame ongoing collective efforts to analyse repression (as the authors prefer) in the Soviet Union, spatially and temporally.

 As the authors note the Gulag remains an incredibly tough subject for geographers. This paper will provide a superb resource to understand the extent so far of efforts to map this system of repression over space and time and the obstacles that remain (and grow).

 I thought the organization of the article makes perfect sense - it brings home just what a huge effort is required to actually identify camp structures below the level of administrative centres. The Memorial database is important on the national level but as the authors' work in Perm shows the efforts of local researchers and NGOs in the regions is truly indispensable to work out how camps were 'dislocated' in Soviet bureaucratic jargon. This is really a huge amount of work which will only get harder and harder to do as Memorial has closed and memorialization of Soviet crimes becomes more and more politicized.

 The authors have years of experience in doing this mapping work, their website is already an important resource and this paper helps to understand their methodologies which others will be able to replicate.

If applicable, is the statistical analysis and its interpretation appropriate?

Yes

Is the study design appropriate and is the work technically sound?

Yes

Is the work clearly and accurately presented and does it engage with the current literature?

Yes

Are the conclusions drawn adequately supported by the results?

Yes

Are sufficient details of methods and analysis provided to allow replication by others?

Yes

Are all the source data and materials underlying the results available?

Yes

Reviewer Expertise:

Criminology, prison sociology, Area Studies (former Soviet Union)

I confirm that I have read this submission and believe that I have an appropriate level of expertise to confirm that it is of an acceptable scientific standard.

# Response

**Published:** 2022-04-30

**Authors:** Judith Pallot

Thank you for this. We are really encouraged by the endorsement for this approach that comes from criminology. While within geography GIS is well understood (although less among historical geographers than those studying current processes), the contribution that mapping and other visualisations can make to research in other disciplinary approaches has yet to take hold. One problem all researchers face (in addition to accessing and putting together the corpus, which you rightly note has suffered a severe blow for those of us interested in Russia since February 24th), is learning the technical skills involved in GIS mapping.

# Peer review: Reviewer response for version 1

**DOI:** 10.21956/openreseurope.15554.r29030

**Published:** 2022-04-21

**Authors:** Susan Grunewald

**Affiliations:** 1 University of Pittsburgh, Pittsburgh, PA, USA

In this article, Judith Pallot and Sofya Gavrilova use geographic information system (GIS) mapping analysis to visualize and analyze the landscapes of Stalinist repression. Following on the example of the Stanford Holocaust Geographies Project, Pallot and Gavrilova combine quantitative and textual data with spatial analysis and visualization with the end goal of countering some myths of the geography of the Soviet gulag and mass repression more broadly. The authors brilliantly present spatial analyses on the national, regional, and individual levels to illustrate the myriad of repressions and punishments in Stalin’s Soviet Russia beyond the widespread trope of confinement in Siberian gulags. Often, as the authors show with the special settlements, different types of forced migration and repression were inter-related. Future work needs to expand the scope of study to investigate the variety of different cases that existed in the Soviet Union’s diverse and far-reaching penal geography.

 The findings of Pallot and Gavrilova are even more impressive given the trials and tribulations of studying a politically charged topic with limited archival access. The bulk of their national level study of Stalinist repression relies on a database originally published by the Russian human rights NGO *Memorial*, which has recently been shut down by the Russian government. Access to regional documents is even more restricted, though local historians, museums, and chapters of *Memorial* helped to contribute the necessary data. As the authors note, however, Soviet penal statistics can be extremely problematic, especially when concerning mortality and morbidity statistics.

 Despite these major issues with the access to and composition of sources, the authors manage to produce and excellent series of maps that illustrate the growth and decline of the Stalinist repression system. The temporal aspect is also another major highlight of their work. For example, the authors build upon other recent scholarship to show that the largest camps appeared first in the European part of Russia and not Siberia. Furthermore, camps opened and closed in different places and at different times depending on needs of the state and the penal system.

 One of the most interesting aspects of the work of Pallot and Gavrilova is their mapping of an anonymized individual’s trajectory through the special settlement system. Drawing inspiration from the Stanford Holocaust Geographies Project, Pallot and Gavrilova again illustrate that the system was dynamic in a variety of ways. Those who went through the special settlements frequently moved based on the state’s need to fulfill economic tasks such as the construction of a paper mill or harvesting timber. Different settlements also featured different target groups of repression, bringing varied populations into contact with each other.

 Scholars of the gulag have frequently noted the existence of the special settlements, which existed within the broader framework of the Stalinist repression and penal camp system. However, they have yet to fully explore the interconnected nature of the two systems. The authors are correct to assert the need to broaden the study of Stalinist mass repressions and develop standards for their visualization along the lines of the Stanford Holocaust Geographies Project. Given the growing prevalence of digitized testimonies of Stalinist repression, the large-scale mapping of individual journeys may be the key to furthering our understanding of the system, especially with a lack of access to archival sources.

 In sum, this article is an important contribution for scholars of the gulag and Stalinist mass repression as well as of those of violent and lethal geographies more broadly. For those unfamiliar with the Soviet case, the work of Pallot and Gavrilova provides key insight to into a major but understudied example of carceral geography. Their work with historical GIS also exemplifies the utility of visualizing data to challenge field assumptions and to provide new avenues for further examination. Finally, their work illustrates the need to study the lived experience within a landscape of mass repression, returning the human element to a potentially otherwise sanitized top-down study of brutality.

If applicable, is the statistical analysis and its interpretation appropriate?

Yes

Is the study design appropriate and is the work technically sound?

Yes

Is the work clearly and accurately presented and does it engage with the current literature?

Yes

Are the conclusions drawn adequately supported by the results?

Yes

Are sufficient details of methods and analysis provided to allow replication by others?

Yes

Are all the source data and materials underlying the results available?

Yes

Reviewer Expertise:

Soviet forced labor, GIS

I confirm that I have read this submission and believe that I have an appropriate level of expertise to confirm that it is of an acceptable scientific standard.

# Response

**Published:** 2022-04-30

**Authors:** Judith Pallot

Thank you for this positive review.  We are aware that there is a vast amount of work yet to be done to fill in all the gaps in representing the different aspects of the Stalinist repressions. The next step would certainly be to produce overlays of your own mapping of the prisoner-of-war camps that are missing from. the maps that accompany the standard histories of the gulag. There are, in addition to the German POWs, the Japanese POW camps in the east, also the location of the places where the labour army was deployed, the correctional labour colonies and others. We really need to try to raise the funding needed to launch a project equivalent to the Stanford Holocaust project. You are right that the problem will be accessing data, especially now but we are fortunate to have some digitized resources. Until the archives become available again, it would be best to focus on the local level and the visualise the sort of personal journeys along the lines we suggest in the article - there are some wonderful sources available in the testimonies of gulag survivors, and the letters that are beginning to surface from deportees and gulag prisoners.
